# p16 promotes proliferation in cervical carcinoma cells through CDK6-HuR-IL1A axis

**DOI:** 10.7150/jca.35479

**Published:** 2020-01-14

**Authors:** Mingzhe Li, Jiong Yang, Kaiyu Liu, Jianming Yang, Xiangwen Zhan, Le Wang, Xiaomeng Shen, Jing Chen, Zebin Mao

**Affiliations:** 1 Department of Biochemistry and Molecular Biology, School of Basic Medical Sciences, Peking University Health Science Center, Beijing, China; 2 Department of Gastroenterology, Peking University Third Hospital, Beijing, China; 3 Department of Immunology, School of Basic Medical Sciences, Tianjin Medical University, Tianjin, China

**Keywords:** Cervical carcinoma, Cyclin-Dependent Kinase Inhibitor p16, Cyclin-Dependent Kinase 6, ELAV-Like Protein 1, Interleukin-1alpha.

## Abstract

The Cyclin-Dependent Kinase Inhibitor p16 (p16) acts as a tumor suppressor in most cells, but for HPV transformed cervical cancer, in which oncoprotein E7 expressed by human papillomavirus (HPV) mediates the degradation of retinoblastoma protein (Rb), p16 exhibits oncogenic activity. Our study was conducted to study the mechanism underling p16 mediated promoting effect of cell proliferation in cervical cancer cell lines. CCK8 assay and EdU incorporation were conducted to evaluate cell proliferation. Loss-of-function assay was used to silence p16 in Ca Ski and SiHa cells. Next, western blot, qPCR, RNA silencing, luciferase activity assay, run-on assay, mRNA stability assay, RNA immunoprecipitation, co-immunoprecipitation Immunofluorescence were performed to examine the interaction between CDK6, HuR, and IL1A mRNA in p16 mediated proliferation promoting effect. Our results showed that: (1) Silencing p16 inhibited the proliferation of cervical cancer cells by decreasing the half-life of IL1A mRNA in CDK6 dependent manner; (2) The stabilization of IL1A mRNA was regulated by HuR which could be inactivated by p16/CDK6 mediated phosphorylation at Ser202; (3) IL1A mediated the oncogenic activity of p16 in cervical carcinoma cell lines. In conclusion, p16 promotes proliferation in cervical carcinoma cells through CDK6-HuR-IL1A axis.

## Introduction

Cervical carcinoma is the commonest cause of death among female in developing countries. The persistent infection of cervix with high risk types of HPV has been considered as a critical cause of cervical carcinoma. In recent years, functional screening programs carried out in developed countries has rendered advanced cervical carcinoma as a relatively rare disease, but in developing countries, the prognosis of cervical carcinoma still remains poor. So, developing new appropriate therapies is quite essential.

As an inhibitor of the Cyclin-Dependent Kinase 4/6 (CDK4/6), the Cyclin-Dependent Kinase Inhibitor p16 (p16) has regulatory roles of CDK4/6 in cell cycle G1 progression 1,which is also known as a vital tumor suppressor. CDKN2A, the gene encoding p16, is deleted or mutated in a wide variety of tumors, such as pancreatic cancer, esophageal cancer, and head and neck cancer. Even though p16 acts as a tumor suppressor in most cellular contexts, as oncoprotein E7 expressed by human papillomavirus (HPV) mediate the degradation of retinoblastoma protein (Rb) 2, a target of the CDK4/6 kinase, the tumor suppressor role of p16 is abolished in HPV transformed cervical cancers. Surprisingly, it has been reported that p16 exhibits oncogenic activity in cervical carcinoma cell lines 3, suggesting that p16 is not only a diagnostic marker of cervical neoplasia, but also is necessary for the survival of cervical carcinoma cells. Because p16 expression is not essential for normal cell proliferation, this difference creates a promising cellular vulnerability for targeting treatments. To develop s therapy against cervical cancer, it is necessary to establish a better understanding of the "oncogenic" activity of p16. In the present research, we studied the "oncogenic" activity of p16 and the molecular mechanisms in cervical cancer cell lines.

## Material and methods

### Cell culture

Ca Ski and SiHa cells were purchased from ATCC (Rockefeller, MD, USA). Cells were cultured as previously described 3.

### Cell proliferation assay and EdU incorporation assay

Cell proliferation assay was performed with CCK8 kit according to the manufacturer's instructions. EdU incorporation assay was performed as previously described 4.

### Plasmid constructs

Plasmid constructs are detailed in the Supporting Information.

### Viral transduction

Infectious virus was produced as previously described 5.

### RNA isolation and Quantitative RT-PCR

Total RNA was extracted using RNeasy Mini Kit (Qiagen). SYBR Green Quantitative RT-PCR (qRT-PCR) analyses were performed as previously described^6^. Taqman qRT-PCR analyses were carried out using THUNDERBIRD Probe qPCR Master Mix according to the manufacturer's instructions. Primer and probe sequences are detailed in the Supporting Information.

All-in-One miRNA qRT-PCR Detection Kit (QP015; GeneCopoeia, I-270 Hi-Tech corridor, MD, USA) was used to evaluate the miRNA expression according to the manufacturer's instructions.

### Protein analysis

Western blot analysis was performed as previously described^6^. Antibodies are detailed in the Supporting Information.

Human IL-1 alpha ELISA Kit (ab100560) was used to detect the concentration of secreted IL-1.

### Immunofluorescence

Cells were fixed, permeabilized and blocked as previously described 7, followed by incubation with anti-HuR antibodies overnight at 4 °C.

### Luciferase activity assay

Ca Ski cells were transfected with 0.45 µg of reporter plasmid together with 0.05 µg of control in a 24-well plate. 36 hours after transfection, luciferase activity was measured with a luminometer (Centro LB 960); firefly luciferase activity was normalized to that of the renilla luciferase activity.

### Cell Cycle Analysis

Cell Cycle Analysis was performed as previously described 6.

### Run-on assay

mRNA stability assay was performed as previously described 8.

### mRNA stability assay

mRNA stability assay was performed as previously described 6 with a minor modification: expression of 18S was used as the control.

### RNA immunoprecipitation

A RIP assay was carried out using the EZ-Magna RIP™ RNA-Binding Protein Immunoprecipitation Kit (Millipore). Antibodies are detailed in the Supporting Information. The precipitated RNAs were purified using RNeasy Mini Kit (Qiagen) and detected by SYBR Green qRT-PCR while microRNAs were purified by the TRIzol reagent (15596; Thermo Fisher Scientific, Waltham, MA, USA) and detected by All-in-One miRNA qRT-PCR Detection Kit (QP015; GeneCopoeia, I-270 Hi-Tech corridor, MD, USA).

### Co-immunoprecipitation

Cells were rinsed and lysed as previously described 7. The lysates were clarified and incubated overnight with IgG, anti-HuR or anti-CDK6 antibody. Next day, bead A/G were added and incubated for 2 hours. After washes with PBS contain 1% glycerol, bound proteins were subjected to Western blot analysis.

### RNA Interference

The sense strands of siRNAs were as follows: si-IL1A#1 5'-GCCTTGTAATTCTAAATGA; si-IL1A#2 5'- GCTATGGCCCACTCCATGA.

### Statistical analysis

Data were presented as mean ± s.d. from at least three independent experiments. The Student's t-test was employed to evaluate the significance. All statistical analyses were performed using Prism 7.0 (GraphPad Software, Inc.), and a P-value < 0.05 was considered significant.

## Results and Discussion

### p16 regulated IL1A expression in cervical cancer cell lines

**Figure 1:**
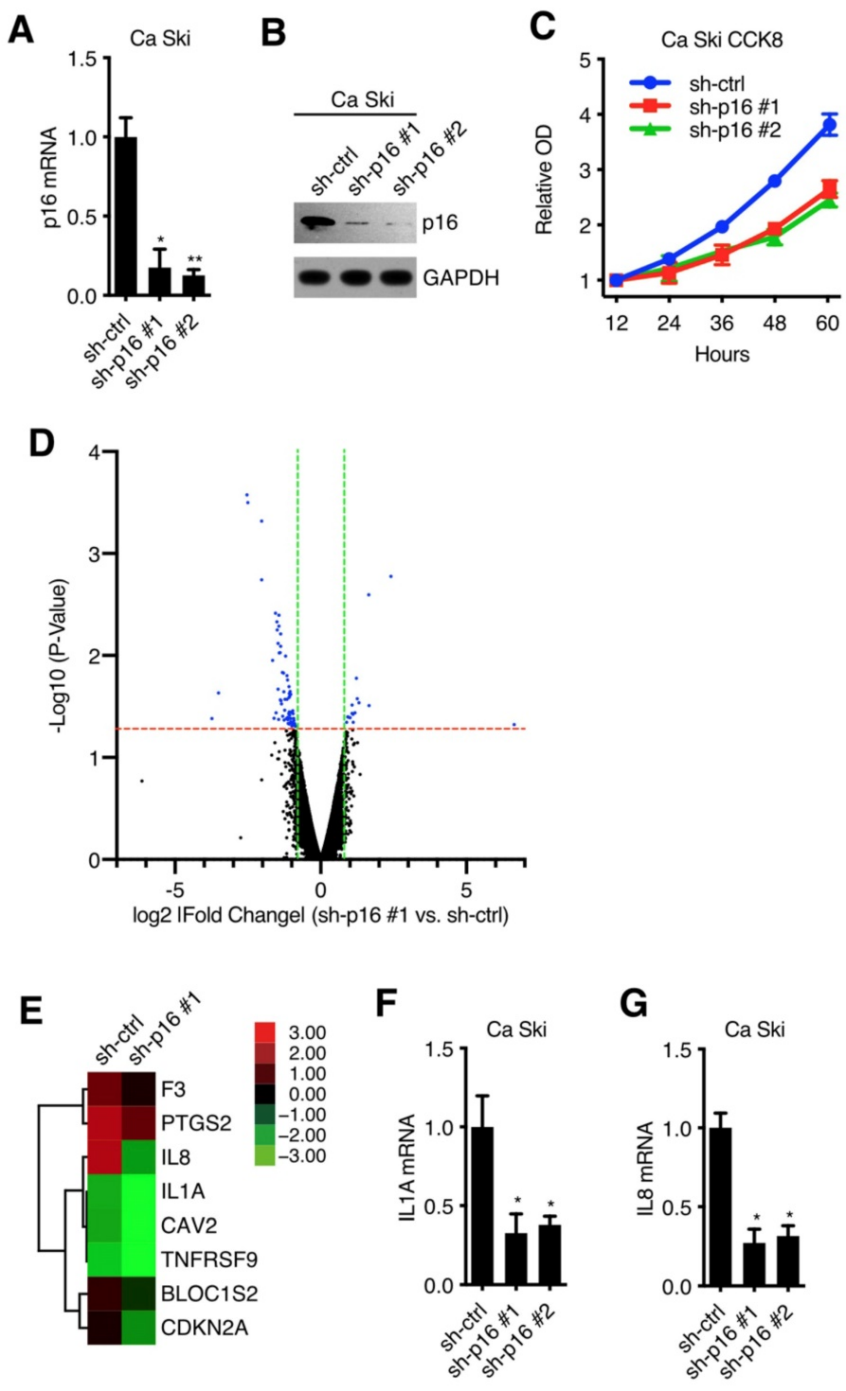
p16 regulated IL1A expression in cervical cancer cell lines: (A) p16 transcript levels of Ca Ski sh-ctrl, Ca Ski sh-p16#1 and Ca Ski sh-p16#2 were determined by SYBR Green qRT-PCR analyses. (B) p16 expression levels of Ca Ski sh-ctrl, Ca Ski sh-p16#1 and Ca Ski sh-p16#2 were determined by Western blotting. GAPDH served as the loading control. (C) Cells proliferation assay of Ca Ski sh-ctrl, Ca Ski sh-p16#1 and Ca Ski sh-p16#2 for 60 hours. (D) A microarray identified 95 genes comparing Ca Ski sh-ctrl and Ca Ski sh-p16#1. The threshold was set as 1.5-fold changes and p < 0.05. (E) Heat maps of 8 genes involved in the regulation of cell proliferation. (F) IL1A transcript levels of Ca Ski sh-ctrl, Ca Ski sh-p16#1 and Ca Ski sh-p16#2 were determined by Taqman qRT-PCR analyses. (G) IL8 transcript levels of Ca Ski sh-ctrl, Ca Ski sh-p16#1 and Ca Ski sh-p16#2 were determined by SYBR Green qRT-PCR analyses.

To verify the oncogenic activity of p16 in cervical carcinoma cell lines, we established 2 kinds of p16 silencing cell lines. Ca Ski and SiHa were transduced with lentivirus particles with different shRNA (against Non-Target control or p16) and subjected to puromycin antibiotic selection, generating Ca Ski sh-ctrl, Ca Ski sh-p16#1, Ca Ski sh-p16#2, SiHa sh-ctrl, SiHa sh-p16#1 and SiHa sh-p16#2 cells. Silencing efficiencies were analyzed by qRT-PCR using SYBR Green and western blot (Figure 1A and B, Figure S1A and B). Figure 1A and Figure S1A showed that two sequences, sh-p16#1 and sh-p16#2, generated over 60% efficiencies in Ca Ski and SiHa, respectively. CCK8 assay showed that p16 knockdown had an impact on the cell viability and proliferation of Ca Ski (Figure 1C) and SiHa cells (Figure S1C), indicating p16 had oncogenic activity in cervical carcinoma cells. Microarray experiment was performed to analyze the gene expression in Ca Ski sh-ctrl and Ca Ski sh-p16#1 (microarray data available at https://drive.google.com/file/d/1ilIO9WrJOT4xhUn_R99aZjJCjh0XnMPJ/view?usp=sharing), showing that 23 genes were upregulated, and 72 genes were downregulated (threshold value fold change > 1.5 and p < 0.05) (Figure 1D). 8 genes including Interleukin-1 alpha (IL1A) and its downstream gene Interleukin-8 (IL8)^9^ that involved in the regulation of cell proliferation were enriched by Gene Ontology analysis (Figure 1E). It has been validated that the expression level of IL1A was associated with the progression and prognosis of cervical cancer 10 and the polymorphism of IL1A was associated with the risk of cervical squamous cell carcinoma 11,12. So, we speculated that IL1A participated in the oncogenic activity of p16. The expression levels of IL1A and IL8 in Ca Ski sh-ctrl, Ca Ski sh-p16#1, Ca Ski sh-p16#2, SiHa sh-ctrl, SiHa sh-p16#1, and SiHa sh-p16#2 were analyzed by qRT-PCR using SYBR Green or Taqman. Figure 1F, 1G, S1D, S1E, S4A, and S4B showed that IL1A and IL8 were downregulated in p16 silencing Ca Ski and SiHa cells, while the expression level of IL1R1 remained the same (Figure S4C and D). The activity of NF-kB was decreased after knocking down p16 in Ca Ski cells (Figure S4E).

### p16 regulated IL1A expression in CDK6 dependent manner

**Figure 2:**
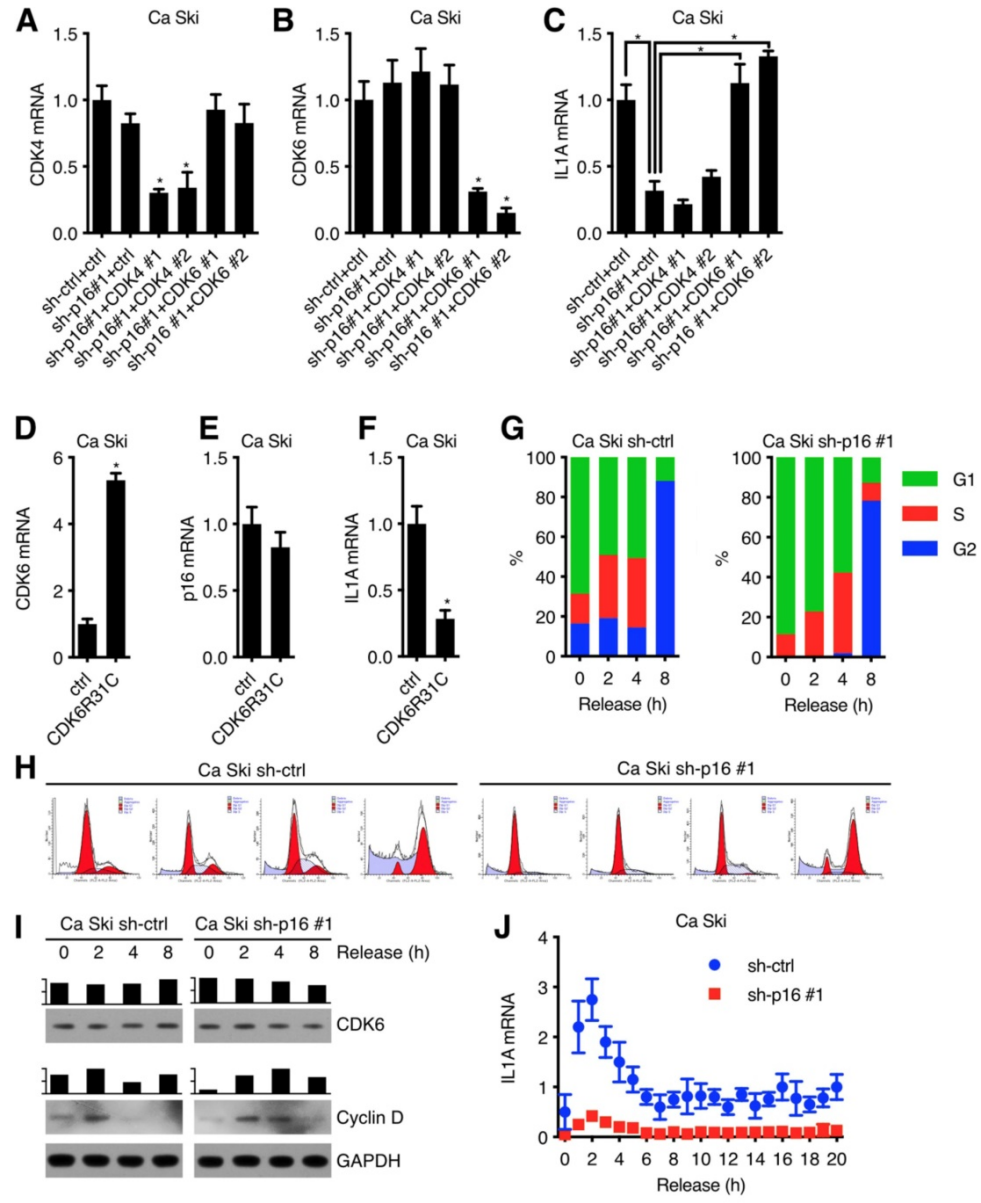
p16 regulated IL1A expression in CDK6 dependent manner: CDK4 (A) and CDK6 (B) transcript levels of Ca Ski sh-ctrl+ctrl, Ca Ski sh-p16#1+ctrl, Ca Ski sh-p16#1+CDK4#1, Ca Ski sh-p16#1+CDK4#2, Ca Ski sh-p16#1+CDK6#1 and Ca Ski sh-p16#1+CDK6#2 were determined by SYBR Green qRT-PCR analyses and IL1A transcript levels (C) were determined by Taqman qRT-PCR analyses. CDK6 (D) and p16 (E) transcript levels of Ca Ski ctrl and Ca Ski CDK6R31C were determined by SYBR Green qRT-PCR analyses and IL1A transcript levels (F) were determined by Taqman qRT-PCR analyses. (G) Cell cycle (H) of synchronized Ca Ski sh-ctrl and Ca Ski sh-p16#1 was measured. (I) CDK6 and Cyclin D expression levels of synchronized Ca Ski sh-ctrl and Ca Ski sh-p16#1 were determined by Western blotting. GAPDH served as the loading control. (J) IL1A transcript levels of synchronized Ca Ski sh-ctrl and Ca Ski sh-p16#1 were determined by Taqman qRT-PCR analyses.

It has been validated that p16 was an inhibitor of the CDK4/6 13-15 with simple protein structure 16,17, so we speculated that p16 upregulated IL1A expression through inhibiting CDK4 or CDK6 kinase activity. To test this hypothesis, Ca Ski sh-ctrl and Ca Ski sh-p16#1 were transduced by lentivirus particles with different shRNAs against Non-Target control, CDK4, CDK6 and then subjected to G418 antibiotic selection to generate Ca Ski sh-ctrl+ctrl, Ca Ski sh-p16#1+ctrl, Ca Ski sh-p16#1+CDK4#1, Ca Ski sh-p16#1+CDK4#2, Ca Ski sh-p16#1+CDK6#1, and Ca Ski sh-p16#1+CDK6#2 cells. The expression levels of CDK4, CDK6 and IL1A were analyzed by qRT-PCR using SYBR Green or Taqman. Figure 2C showed that silencing CDK6 rescued IL1A expression, suggesting that p16 upregulated IL1A through CDK6. To determine whether the downregulation of IL1A depended on CDK6 kinase activity or not^18^,^19^, we designed an experiment to investigate whether the overexpression of CDK6R31C, which was immune to the inhibition of p16, could downregulate IL1A expression. Figure 2F showed that overexpression of mutant CDK6 downregulated IL1A expression. Given that CDK6 kinase activity was detected in the G1 phase of cell cycle and associated with Cyclin D, we speculated that IL1A expression was influenced by cell cycle progression. Ca Ski sh-ctrl and Ca Ski sh-p16#1 were synchronized at G1 phase by thymidine (TdR) double blocking. Figure 2G, H and I showed that after release, synchronized cells entered cell cycle with DNA duplication and the classic change of CDK6 and G1 marker (Cyclin D). Figure 2J showed that compared with control group, IL1A mRNA level increased dramatically in Ca Ski sh-p16#1 at 2 h, indicating that IL1A mRNA level was negatively corelated with CDK6 kinase activity which was highly depended on Cyclin D expression. All of these data pointed that p16 regulated IL1A mRNA level through inhibiting CDK6 kinase activity.

### p16/CDK6 regulated IL1A mRNA stability

**Figure 3:**
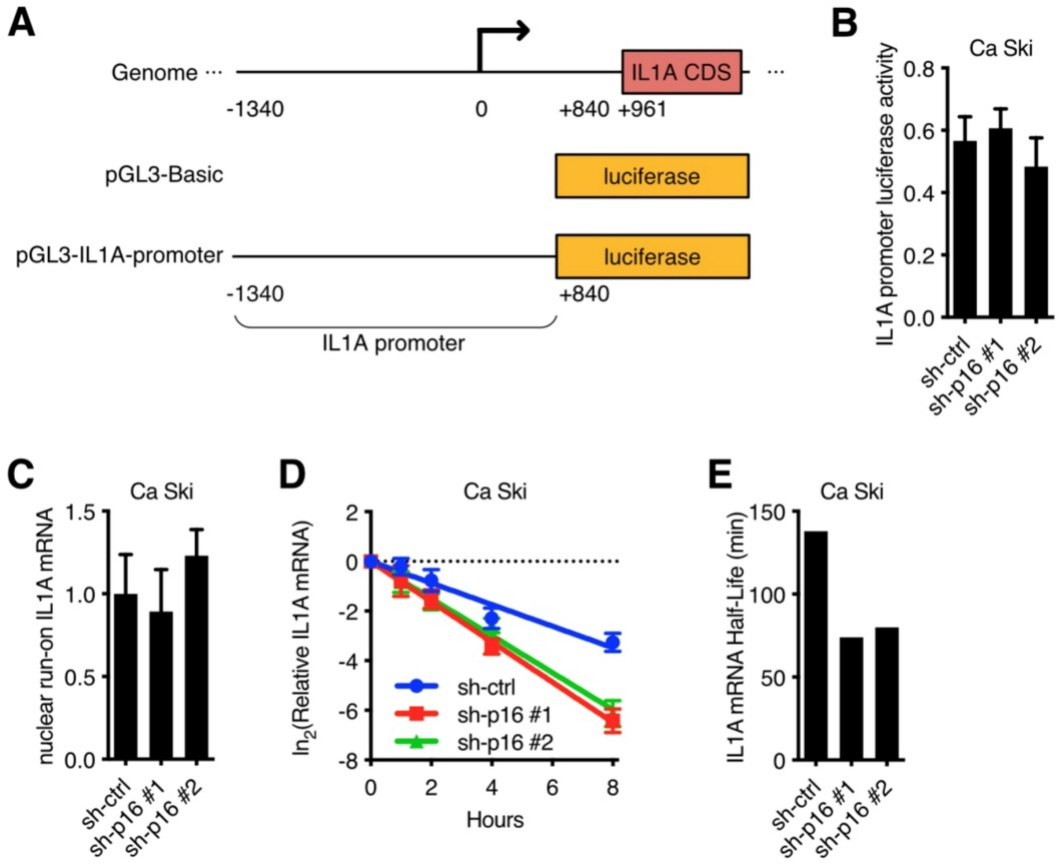
p16/CDK6 regulated IL1A mRNA stability: (A) A schematic diagram of IL1A promoter luciferase was constructed. (B) The activity of IL1A promoter in Ca Ski sh-ctrl, Ca Ski sh-p16#1 and Ca Ski sh-p16#2 was analyzed by luciferase-based reporter assay. (C) Nuclear run-on assay of Ca Ski sh-ctrl, Ca Ski sh-p16#1 and Ca Ski sh-p16#2. (D) The stability of endogenous IL1A mRNA was influenced by p16 knockdown. (E) The half-life of IL1A mRNA in Ca Ski sh-ctrl, Ca Ski sh-p16#1 and Ca Ski sh-p16#2 were calculated.

To further investigate the mechanism underlying the regulation of p16 to IL1A mRNA in cervical cancer cell lines, IL1A promoter luciferase activity assay was performed. A 1.4 kb IL1A promoter was fused to the luciferase reporter gene (Figure 3A) and then transfected into Ca Ski sh-ctrl, Ca Ski sh-p16#1 and Ca Ski sh-p16#2. Silencing p16 showed no effect on the luciferase activity of 1.4 kb IL1A promoter (Figure 3B). Similar results were observed in SiHa cells (Figure S3A). Because long-range cis-element or epigenetic modification might regulate IL1A promoter activity, a nuclear run-on assay was performed. Figure 3C showed that the transcription initiation rate of IL1A promoter in Ca Ski sh-ctrl, Ca Ski sh-p16#1 and Ca Ski sh-p16#2 had no difference. Similar results were observed in SiHa cells (Figure S3B). As IL1A expression changed at RNA level, these results suggested that the regulation of p16/CDK6 to IL1A happened at post-transcriptional level. To further evaluate the effect of p16 on IL1A regulation, a mRNA stability assay with actinomycin D was performed in Ca Ski sh-ctrl, Ca Ski sh-p16#1 and Ca Ski sh-p16#2. Figure 3D and E showed that silencing p16 resulted in a decrease of the half-life of IL1A mRNA. Similar results were also observed in SiHa cells (Figure S3C and D). Together, these data indicated that p16/CDK6 regulated IL1A in post-transcriptional level by controlling its mRNA stability.

### HuR participated in the regulation of IL1A mRNA stability

**Figure 4:**
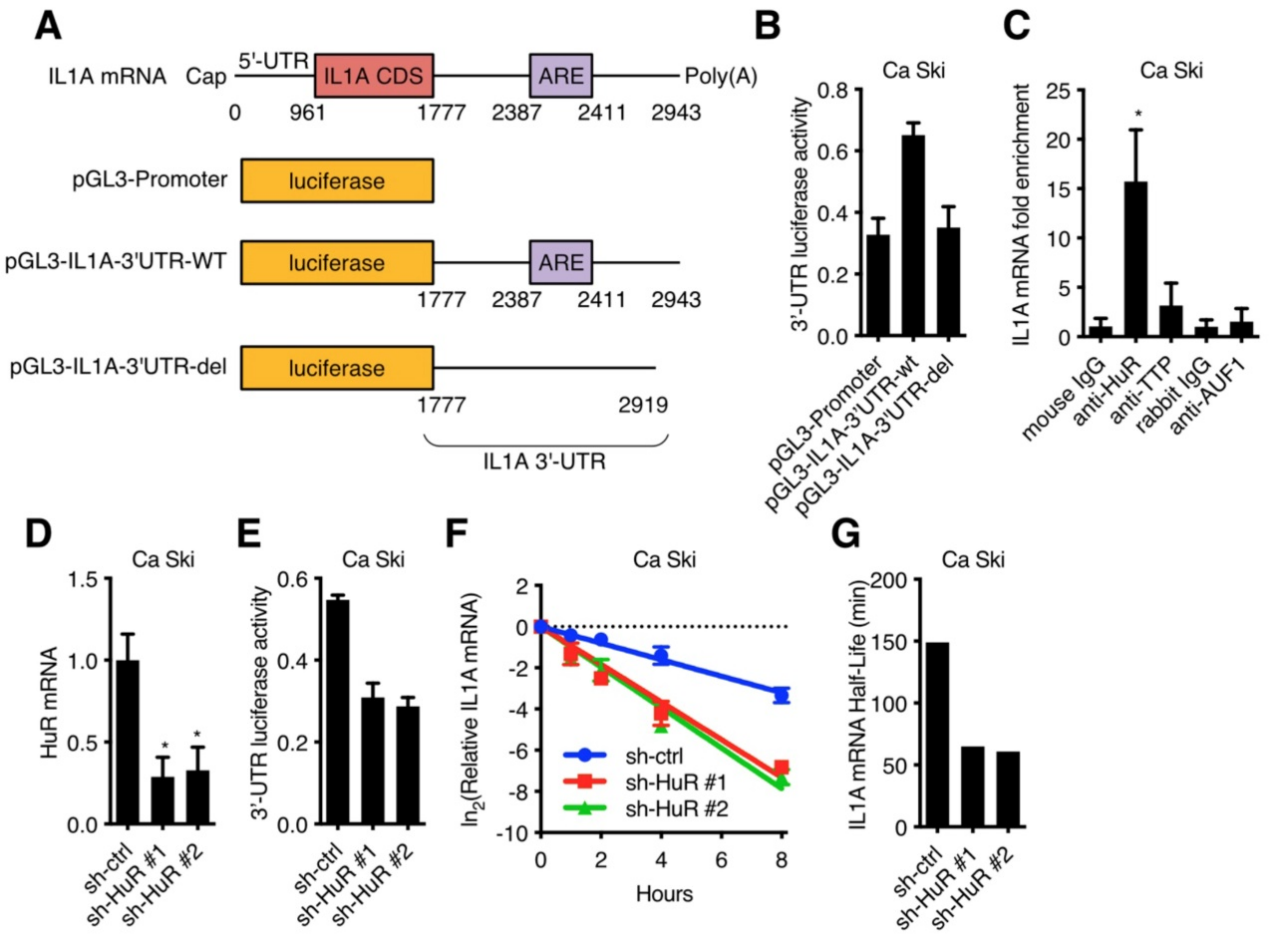
HuR was involved in IL1A mRNA stability regulation: (A) A schematic diagram of IL1A 3'-UTR luciferase was constructed. (B) The activity of luciferase-based reporter of the IL1A 3'-UTR in Ca Ski cells was analyzed. (C) The interaction between RNA binding proteins and IL1A mRNAs was examined by RNA immunoprecipitation. Mouse IgG was included as a negative control. (D) HuR transcript levels of Ca Ski sh-ctrl, Ca Ski sh-HuR#1 and Ca Ski sh-HuR#2 were determined by SYBR Green qRT-PCR analyses. (E) The activity of luciferase-based reporter of the IL1A 3'-UTR in Ca Ski sh-ctrl, Ca Ski sh-HuR#1 and Ca Ski sh-HuR#2 was analyzed. (F) The stability of endogenous IL1A mRNA was affected by HuR knockdown. (G) The half-life of IL1A mRNA in Ca Ski sh-ctrl, Ca Ski sh-HuR#1 and Ca Ski sh-HuR#2 were calculated.

It has been reported that RNA binding protein could recognize target RNA which contains ARE (AU rich element) and regulate its stability 20,21. 3'-UTR luciferase activity assay was performed in Ca Ski to determine whether IL1A mRNA was stabilized by RNA binding proteins. IL1A 3'-UTR-wt or IL1A 3'-UTR-del (without ARE) were fused to the luciferase reporter gene (Figure 4A) and transfected into Ca Ski. IL1A 3'-UTR without ARE showed similar luciferase activity as control, while wild-type IL1A 3'-UTR increased luciferase activity, suggesting that RNA binding proteins increased IL1A stability (Figure 4B). IL1A mRNA binding proteins were screened in Ca Ski by RNA immunoprecipitation (RIP) and qRT-PCR. Figure 4C showed that anti-HuR antibody enriched IL1A mRNA, suggesting ELAV-Like Protein 1 (HuR) influenced IL1A mRNA stability. It has been reported that the RNA-binding protein HuR could stabilize or destabilize target mRNAs 22. To verify whether HuR regulated IL1A mRNA in Ca Ski cell lines, HuR silencing Ca Ski cell lines were established. Ca Ski were infected with lentivirus particles with different shRNAs against Non-Target control or HuR and then subjected to puromycin antibiotic selection for Ca Ski sh-ctrl, Ca Ski sh-HuR#1, Ca Ski sh-HuR#2. Silencing efficiencies were analyzed by qRT-PCR using SYBR Green (Figure 4D). Similar results were shown in SiHa cells (Figure S3E). Figure 4D showed that two sequences, sh-HuR#1 and sh-HuR#2, generated over 50% efficiencies in Ca Ski. 3'-UTR luciferase activity assay was performed in Ca Ski sh-ctrl, Ca Ski sh-HuR#1, Ca Ski sh-HuR#2. Silencing HuR decreased IL1A 3'-UTR luciferase activity, suggesting that HuR stabilized IL1A mRNA in Ca Ski. Then the half-life of IL1A was measured in Ca Ski sh-ctrl, Ca Ski sh-HuR#1 and Ca Ski sh-HuR#2. Figure 4F, 4G, S3F, S3G, and S4F showed that the stability of IL1a mRNA in Ca Ski and SiHa cells was decreased after silencing HuR and IL8 expression was also decreased. These results supported the argument that HuR stabilized IL1A mRNA in cervical cancer cell lines.

### CDK6 phosphorylated HuR at Ser202

**Figure 5:**
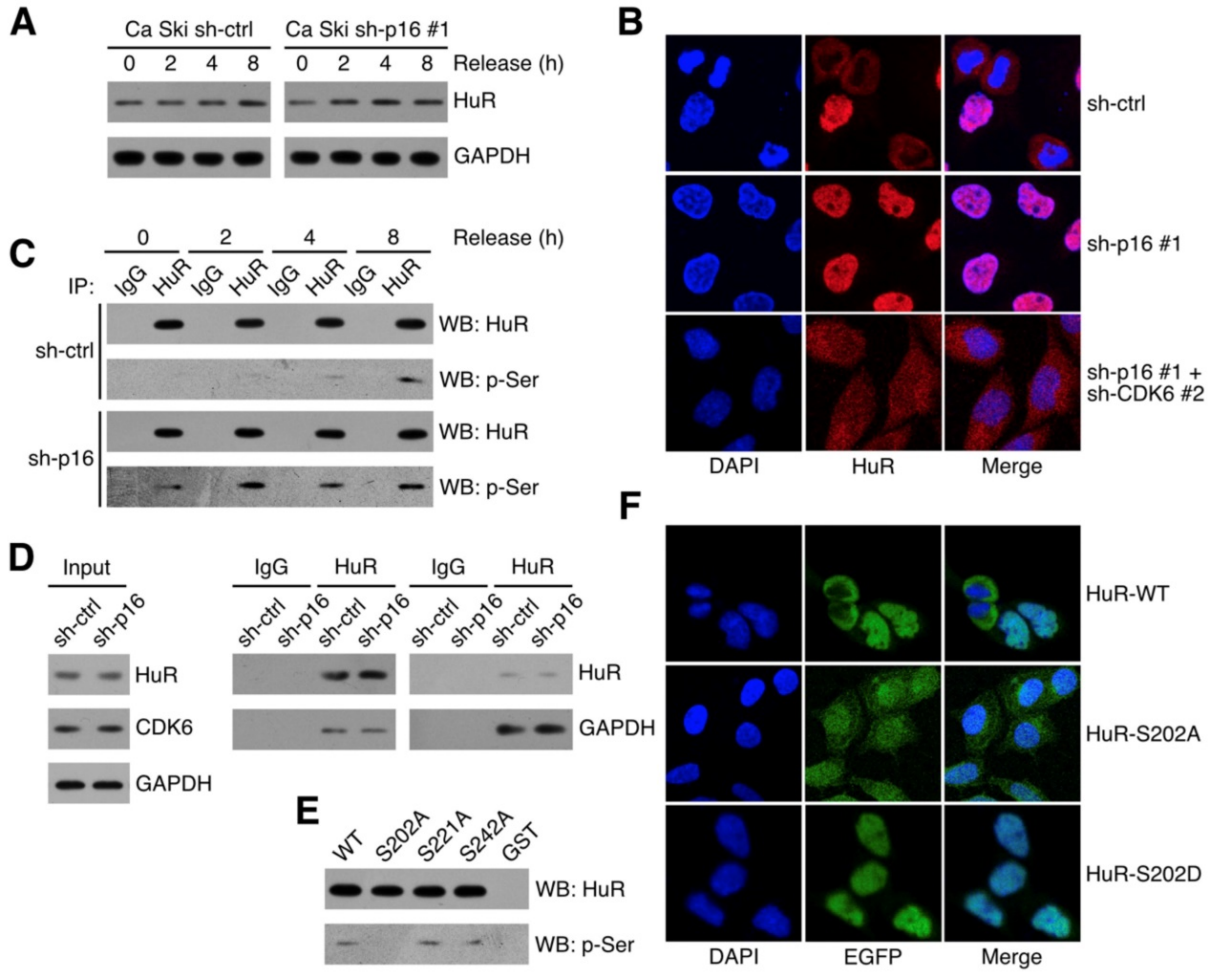
CDK6 phosphorylated HuR at Ser202: (A) HuR expression level in synchronized Ca Ski sh-ctrl and Ca Ski sh-p16#1 were determined by Western blotting. (B) Confocal microscopy images of Ca Ski sh-ctrl, Ca Ski sh-p16#1 and Ca Ski sh-p16#1+CDK6#2. HuR (red) and DAPI staining (blue) was visualized by immunfluorescence. (C) The phosphorylation level of HuR in synchronized Ca Ski sh-ctrl and Ca Ski sh-p16#1 were examed by Co-Immunoprecipitation. (D) Immunoprecipitation and western blot analysis were performed by using the indicated antibodies in the panel. (E) In vitro kinase assay was performed to examine the phosphorylation of HuR by CDK6+CCND1. The phosphorylation level of wild-type HuR (WT) or mutant HuR (S202A, S221A, and S242A) were examined by Co-Immunoprecipitation. (F) The localization of wild-type (top panel) or mutant EGFP-HuR (S202A, middle panel, or S202S, bottom panel) was showed by confocal assay.

It has been reported that HuR stabilized RNAs in the cytoplasm, so the subcellular localization of HuR was critical to its function 23,24. Figure 5A showed that HuR expressed in synchronized Ca Ski sh-ctrl and Ca Ski sh-p16#1. Figure 5B showed that cytoplasmic HuR signals decreased in p16 silencing Ca Ski cells and knocking down of CDK6 could rescue cytoplasmic HuR signals. Many studies demonstrated that HuR subcellular localization was regulated by phosphorylation 25-28, we hypothesized that the phosphorylation of HuR was mediated by p16/CDK6 in cervical cancer cells. To analysis HuR phosphorylation level, co-immunoprecipitation of HuR followed by western was performed in synchronized Ca Ski sh-ctrl and Ca Ski sh-p16#1. Figure 5C showed that the phosphorylation of HuR was affected by CDK6 kinase activity and the silence of p16. Co-immunoprecipitation of HuR followed by western analysis indicated that HuR binded with CDK6, irrespective of p16 presence (Figure 3A). In reciprocal immunoprecipitations, CDK6 was observed binding with HuR, prompting HuR as a potential target of CDK6. The subcellular localization of HuR was regulated by the phosphorylation of hinge region 29. Each of the serine residues in this region was mutated to alanine to generate HuR-S202A, HuR-S221A and HuR-S242. To identify the phosphorylation site of CDK6, in vitro kinase assay was performed by incubating recombinant human CDK6+CCND1 protein with GST-tagged HuR. Figure 5E showed that CDK phosphorylated HuR in vitro, supporting the argument that p16/CDK6 directly regulated HuR. The S202A mutation blocked the phosphorylation of HuR, while mutations at S221A and S242A did not affect HuR phosphorylation (Figure 5E), indicating that HuR was phosphorylated by CDK6 at S202. To further verify the subcellular localization of S202 phosphorylated HuR in Ca Ski cell lines, EGFP-tagged HuR-WT, HuR-S202A and HuR-S202D were ectopically expressed in Ca Ski cell lines respectively. Figure 5F showed that cytoplasmic EGFP signals got increased in EGFP-HuR-S202A expressing cells and decreased in EGFP-HuR-S202D expressing cells, consistent with the observation in HeLa cells 28.

### IL1A mediated the oncogenic activity of p16 in cervical carcinoma cell lines

**Figure 6:**
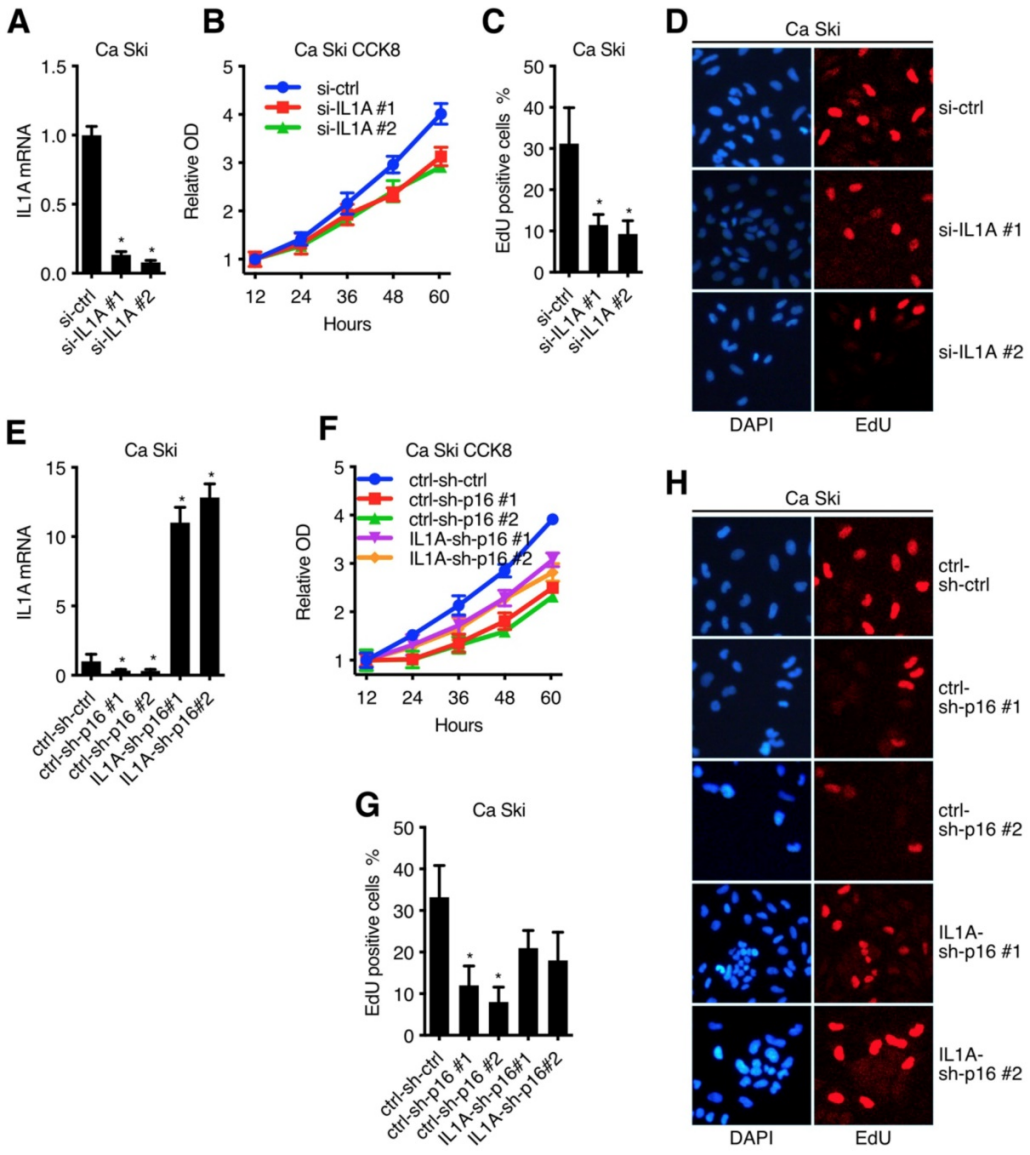
IL1A mediated the oncogenic activity of p16 in cervical carcinoma cell lines: (A) Ca Ski Cells were transfected with independent siRNA against Non-Target control or IL1A. IL1A transcript levels were determined by Taqman qRT-PCR analyses. IL1A knockdown affected cell viability (B) and proliferation (C)(D) of Ca Ski. (E) IL1A transcript levels of Ca Ski ctrl-sh-ctrl, Ca Ski ctrl-sh-p16#1, Ca Ski ctrl-sh-p16#2, Ca Ski IL1A-sh-p16#1 and Ca Ski IL1A-sh-p16#2 were determined by Taqman qRT-PCR analysis. IL1A overexpressing affected the cell viability (F) and proliferation (D)(H) of p16 silencing Ca Ski.

After clarified the signal transduction from p16 to IL1A in Ca Ski, we went on to discuss the oncogenic activity of IL1A^30^. Figure 6A and Figure S2A showed that two sequences, si-IL1A#1 and si-IL1A#2, generated over 70% efficiencies in Ca Ski and SiHa, respectively. CCK8 assay and EdU incorporation are showed that p16 knockdown of got an impact on cell viability and proliferation of both Ca Ski (Figure 6B) and SiHa (Figure S2B), indicating the oncogenic activity of IL1A. To complement the findings with IL1A siRNAs, rescue experiment was conducted by overexpressing IL1a in p16 silencing cells. Ca Ski sh-ctrl, Ca Ski sh-p16#1 and Ca Ski sh-p16#1 were transduced with lentivirus particles expressing EGFP or IL1A and subjected to G418 antibiotic selection to establish Ca Ski ctrl-sh-ctrl, Ca Ski ctrl-sh-p16#1, Ca Ski ctrl-sh-p16#2, Ca Ski IL1A-sh-p16#1 and Ca Ski IL1A-sh-p16#2. CCK8 assay and EdU incorporation showed that overexpressing IL1A rescued the cell viability and proliferation of p16 silencing Ca Ski (Figure 6F, G and H). Similar results were observed in SiHa (Figure S2C and D).

## Discussion

IL1A plays a pivotal role in cervical carcinogenesis and progression. Pu Y et al. provided evidence after conducted a case-control study involving 319 patients with cervical cancer and 424 healthy control women, illustrating that among 2 polymorphisms of *IL1A* gene, the allele I of rs3783553 may related with reduced cervical carcinogenesis risk, reduced susceptibility advancing to stage II-III or developing non-squamous cell carcinoma 11. Huang J et al. PCR-genotyped the *IL1A* rs3783553 polymorphism in a clinical study including 235 patients with cervical squamous cell carcinoma and 326 controls, demonstrating that the ins/ins genotype reduced the risk to develop cervical squamous cell carcinoma 12. Those data pointed out rs3783553 increased cervical carcinogenesis predisposition. As a TICA/-SNP located in IL1A 3'-UTR, rs3783553 increased IL1A expression level by eliminating the targeting point of miR-122 and miR-378 (Figure S5B and C). Aligned with those studies, our research validated IL1A promoted the proliferation of both Ca Ski and SiHa cells. It has been proved that rs3783553 increased the onset risk of breast cancer 31, prostatic cancer 32, ovarian cancer 33, and non-small-cell lung cancer (NSCLC) 34. Compared with pulmonary tuberculosis group, NSCLC patients showed more abundant IL1A in pleural effusion 35. HPV infection was suspected in all those tumor tissues, prompting that the tumor-promoting effect of IL1A may due to HPV-mediated rearrangement of signal pathway in host cells.

Belonging to immunoglobin superfamily, IL-1 receptor (IL-1R) is abundantly expressed in various types of cells. Type I IL-1 receptor (IL-1RI) is a signal receptor that can be activated by IL-1 upon ligand binding and subsequently recruits IL-1R accessory proteins (IL-1RcP), forming signaling receptor complex which activates NF-κB pathway to upregulate downstream molecules including IL-8 36-38. This indicates decrease of IL1A may explain the downregulation of IL8 showed in qPCR and Gene Chip. So, we put exploring the mechanism regulating IL1A at the priority in our research.

CDK6 was identified as an inducible member of CDK family after the discovery of CDK4. For a long time, CDK6 was considered as a homologous protein, functionally redundant with CDK4 39-42. Embryos of *Cdk6* or *Cdk4* knock out mice died at the late stage of embryonic development because of the hematopoietic-deficiency caused anemia. This indicated CDK4 and CDK6 may exhibit the same function. But the phenotypes of *Cdk6* and *Cdk4* knock out mice were different: loss of *Cdk4* expression resulted in reduction of both beta-islet pancreatic cells and pituitary endocrine cells 43,44, while loss of *Cdk6* expression caused deficiency of T-cell function 45,46. Although CDK4 and CDK6 were expressed in all those tissues 39,47,48, loss of *Cdk6* and *Cdk4* influenced different cell types. In this article, our data indicated CDK6, not CDK4 participated in the modulation of IL1A expression.

Overexpression of CDK4 or CDK6 which was insensitive to inhibition by p16 could suppress cell proliferation in HPV positive cells. McLaughlin-Drubin et al. proposed a hypothesis to explain: in pRb-inactivated cervical cancer cells, there were other relevant CDK4/CDK6 substrates that could be phosphorylated to influence cell proliferation 3. Our presented research revealed HuR was exactly this kind of substrate. Recent study identified CDK6 as a transcriptional regulatory factor 19,49,50. K43M mutation validated that the transcription promoting effect of CDK6 had nothing to do with its kinase activity 18,51. As a transcriptional regulatory factor, CDK6 mediated cell-stimulation effect of IL-1 jointly with NF-κB in cervical cancer cells 9. Analysis pointed out that 44% of CDK6 gene binding sites were close to the gene binding site of NF-κB. Without using kinase activity, CDK6 could bind the promoter of IL8, interact and recruit p65 52. This may explain why overexpressing IL1A could not fully rescue the proliferation inhibiting effect caused by knocking down p16: after knocking down p16, CDK6 got a relief from suppression, which then could use not only kinase activity but also transcriptional regulatory ability to synergistically inhibit proliferation of cervical cancer cells.

There are interactions between RNA binding proteins and microRNAs. Bhattacharyya SN et al. found that HuR could relief microRNA miR-122-induced inhibition in carcinoma cells by binding cationic amino acid transporter 1 (CAT-1) mRNA under different stress conditions 53,54. It is noteworthy that SNP rs3783553 of IL1A increased the risk of cervical carcinoma by eliminating targeting points of miR-122. HuR binds and stabilizes the mRNA of IL1A, which prompting that HuR and miR-122 may interact with each other to regulate the expression of IL1A in cervical carcinogenesis and progression.

## Conclusions

Our current study showed that: (1) Silencing p16 inhibited the proliferation of cervical cancer cells by decreasing the half-life of IL1A mRNA in CDK6 dependent manner; (2) The stabilization of IL1A mRNA was regulated by HuR which could be inactivated by p16/CDK6 mediated phosphorylation at Ser202; (3) IL1A mediated the oncogenic activity of p16 in cervical carcinoma cell lines. In conclusion, p16 promotes proliferation in cervical carcinoma cells through CDK6-HuR-IL1A axis.

## Supplementary Material

Supplementary figures.

### Acknowledgements

This research did not receive any specific grant from funding agencies in the public, commercial, or not-for-profit sectors.

## Abbreviations

p16: Cyclin-Dependent Kinase Inhibitor p16
HPV: human papillomavirus
CDK4/6: Cyclin-Dependent Kinase 4/6
Rb: retinoblastoma protein
qRT-PCR: Quantitative RT-PCR
TdR: thymidine
ARE: AU rich element
RIP: RNA immunoprecipitation
HuR: ELAV-Like Protein 1
NSCLC: non-small-cell lung cancer
IL-1R: IL-1 receptor
IL-1RI: Type I IL-1 receptor
IL-1RcP: IL-1R accessory proteins
CAT-1: cationic amino acid transporter 1
