# Supplementary material for: p16 promotes proliferation in cervical carcinoma cells through CDK6-HuR-IL1A axis
Source: J Cancer. 2020 Jan 14;11(6):1457–67. doi: 10.7150/jca.35479 (PMC6995400; doi:10.7150/jca.35479)
Supplement: Supplementary file 1 — Supplementary figures. [file jcav11p1457s1.pdf]

Supplementary Materials

Plasmid constructs

shRNA lentivirus plasmid was created with the following sense shRNA sequences into LentiLox 3.7 (pLL3.7). sh-ctrl 5'-TTCTCCGAACGTGTCACGT; sh-p16#1 5'-GGCACCAGAGGCAGTAACCAT; sh-p16#2 5'-GCCCAACGCACCGAATAGT; sh-CDK4#1 5'-GAGATTACTTTGCTGCCTTAA; sh-CDK4#2 5'-GTTCTTCTGCAGTCCACATAT; sh-CDK6#1 5'-GCAGAAATGTTTCGTAGAA; sh-CDK6 #2 5'-GCAAAGACCTACTTCTGAA; sh-HuR#1 5'-GCAGCATTGGTGAAGTTGAAT and sh-HuR#2 5'-GCGACTTCAACACCAACAA.

CDK6R31C expressing lentivirus plasmid was created as follow: the cDNA for CDK6 was amplified by PCR and subcloned into the NotI and BamHI recognition sites of pITA vector. Primer sets used as follows: 5'-ataagaatGCGGCCGCaATGGAGAAGGACGGCCTG and 5'-cgcGGATCCctAGGCTGTATTCAGCTCCGAG. The underlined sequences are the NotI and BamHI recognition sites. 5'-AAGGTGTTCAAGGCCtGCGACTT and 5'-aGGCCTTGAACACCTTCCCATAGG was used to create the R31C mutant. pGEX-HuR-WT was created as follows: the cDNA for HuR was amplified by PCR and subcloned into pGEM-T Easy Vector. The S202A mutagenic primer: 5'-CTCGCAGCTGTACCACgCGCCAGCGCGAC and 5′-cGTGGTACAGCTGCGAGAGGAGTGCCACGTTT; S221A mutagenic primer: 5'-GCAGAGATTCAGGTTCgCCCCCATGGGCGTC and 5'-cGAACCTGAATCTCTGCGCCTGGTGGTGAAC; S242A mutagenic primer: 5'-GCCAGGAAACGCCTCCgCCGGCTGGTGCAT and 5'-cGGAGGCGTTTCCTGGCACGTTGACGCCAGA were used to create mutant construct. The lower-case bases are the mutated bases. 5'-5'cgGAATTCCatgagcAATGGTTATGAAGACCACATGGC and 5'-ccgCTCGAGTCtcATTTGTGGGACTTGTTGGTTTTG were used to amplify wild-type and mutant HuR. The underlined sequences are the EcoRI and XhoI recognition sites. The PCR products were subcloned into pGEX-4T-3 (GE Healthcare Life Science).

5'-CTCGCAGCTGTACCACgatCCAGCGCGACG and 5'-atcGTGGTACAGCTGCGAGAGGAGTGCCACGTTT were used to create S202D mutant in HuR. To obtain EGFP-tagged HuR plasmids, wild-type and mutant HuR were amplified by PCR and subcloned into the XhoI and EcoRI sites of the pEGFP-C1. Primer sets used as follows: 5'-ccgCTCGAGctatgtctAATGGTTATGAAGACCACATGGC and 5'-cgGAATTCttATTTGTGGGACTTGTTGGTTTTG. 5'-ataagaatGCGGCCGCTGGTGAGCAAGGGCGAG and 5'-ccgGTTAACttATTTGTGGGACTTGTTGGTTTTG were used to subclone the coding sequence of the EGFP-HuR fusion protein into the NotI and BamHI recognition sites of pITA vector.

5'-cggGGTACCCTGAAGCCACCCAAAGTCCCTGTCA and 5'-ctaGCTAGCACAAGTGCGTCGTCAAAACGGGGAA were used to amplify the IL1A promoter. The underlined sequences are the KpnI and NheI recognition sites. The PCR products of the promoter were subcloned into the pGL3-basic plasmid (Promega). IL1A 3'-UTR luciferase constructs were created as follow: The 3'-UTR of IL1A was amplified by PCR and subcloned into the XbaI site of the pGL3-Promoter. Primer sets used as follows: 5'- gcTCTAGAGTCTGGAGTCTCACTTGTCTCACT and 5'-gcTCTAGAGTCAGAGAATTTTGTTGCAAGC. 5'-CGAAATGTTATTTTTTAATTAT/ATTTAAGATAATTATA and 5'-ATTAAAAAATAACATTTCGTGCTTTG were used to remove ARE element.

IL1A expressing lentivirus plasmid was created as follow: the cDNA for IL1A was amplified by PCR and subcloned into the XbaI and BsrGI recognition sites of the iDuet vector. Primer sets used as follows: 5'- tgcTCTAGATGGCCAAAGTTCCAGACAT and 5'-tcgTGTACACTACGCCTGGTTTTCCAGTATC. The underlined sequences are the NotI and BamHI recognition sites.

SYBR Green qRT-PCR analyses

Primer sets used as follows: GAPDH 5'-ACGGATTTGG TCGTATTGGG and 5'-TGATTTTGGAGGGATCTCGC; 18s RNA 5'-GTAACCCGTTGAACCCCATT and 5'-CCATCCAATCGGTAGTAGCG; IL8 5'-CACTGCGCCAACACAGAAAT and 5'-GCTTGAAGTTTCACTGGCATC; CDK4 5'-AGTGTTGGCTGTATCTTTGCAG and 5'-CCATCTCAGGTACCACCGAC; CDK6 5'-GGACGTGATTGGACTCCCAG and 5'-ACAGGGCACTGTAGGCAGAT; AUF1 5'-TATCCAGGCGAGGTGGTCAT and 5'-TATTAGCAGGTGGCAGGAGC; HuR 5'-CACCAGGCGCAGAGATTCA and 5'-TGGTCACAAAGCCAAACCCT; TTP 5'-CATGGCCAACCGTTACACCA and 5'-TCCATGGTCGGATGGCAC. IL1R1 5'-GTCTTGCCTGAGGTCTTGGA and 5'-AGCCAGCTGAAGCCTGATG.

Taqman qRT-PCR analyses

Primer sets and probes used as follows, lower case stands for Locked Nucleic Acid (LNA) bases: GAPDH 5'-ACAACAGCCTCAAGATCATCAGCAAT, 5'-GTCCTTCCACGATACCAAAGTTGTCA and 5'-FAM-CCTcCTGcACCaCCaACTGC-TAMRA; p16 5'-CATAGATGCCGCGGAAGGT, 5'-CCCGAGGTTTCTCAGAGCCT and 5'-FAM-CCGaTtGaAaGaACCaGAGaG-TAMRA; IL1A 5'-AGTAGCAACCAACGGGAAGG, 5'-AAGGTGCTGACCTAGGCTTG and 5'-FAM-ATCGcCaATGaCTCaGAGgAAGA-TAMRA.

Protein analysis

The primary antibodies used were for p16 (1:500 sc-759; Santa Cruz), CDK6 (1:500 sc-271364; Santa Cruz), GAPDH (1:5000 E021010; EarthOx), AUF1 (1:800 ab50692; Abcam), HuR (1:400 sc-5261; Santa Cruz), TTP (1:400 sc-14030; Santa Cruz), Cyclin D (1:500 sc-753; Santa Cruz) and p-Ser (1:125 ab9332; Abcam); The secondary antibodies used were HRP, Goat Anti-Mouse IgG(H+L) (1:5000 E030110-02; EarthOx) and Peroxidase AffiniPure Goat Anti-Rabbit IgG (H+L) (1:5000 98261; Jackson).

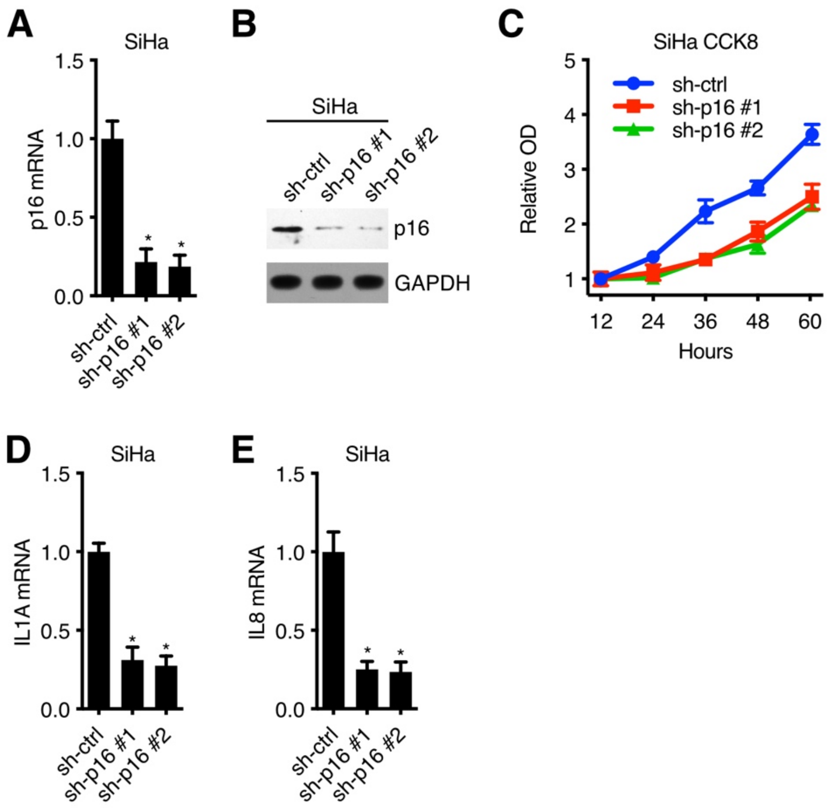

Figure S1. p16 regulated IL1A expression in SiHa cells: (**A**) p16 transcript levels of SiHa sh-ctrl, SiHa sh-p16#1 and SiHa sh-p16#2 were determined by SYBR Green qRT-PCR analyses. (**B**) p16 expression levels of SiHa sh-ctrl, SiHa sh-p16#1 and SiHa sh-p16#2 were determined by Western blotting. GAPDH served as a loading control. (**C**) Cells proliferation assay of SiHa sh-ctrl, SiHa sh-p16#1 and SiHa sh-p16#2 for 60 hours. (**D**) IL1A transcript levels of SiHa sh-ctrl, SiHa sh-p16#1 and SiHa sh-p16#2 were determined by Taqman qRT-PCR analyses. (**E**) IL8 transcript levels of SiHa sh-ctrl, SiHa sh-p16#1 and SiHa sh-p16#2 were determined by SYBR Green qRT-PCR analyses.

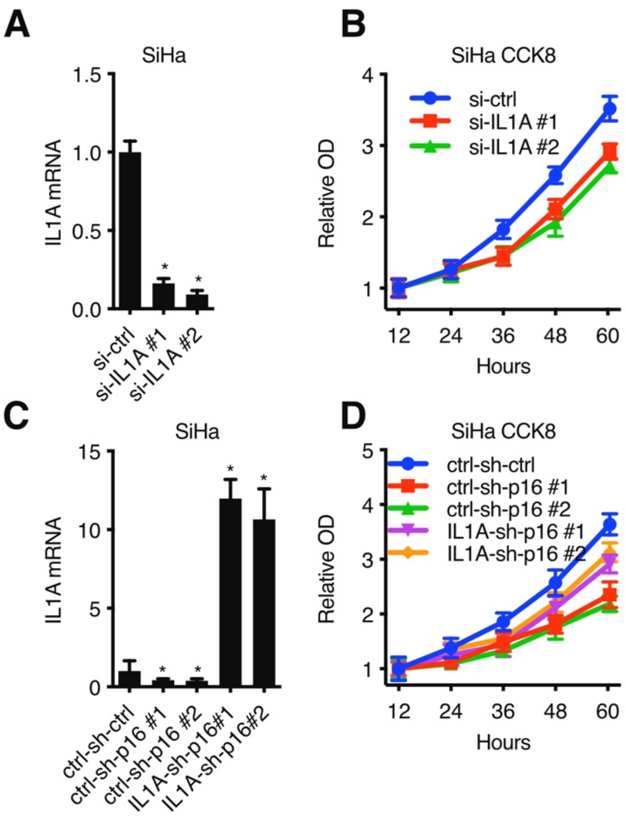

Figure S2. IL1A mediated the oncogenic activity of p16 in SiHa cells: (**A**) SiHa Cells were transfected with independent siRNA against Non-Target control or IL1A. IL1A transcript levels were determined by Taqman qRT-PCR analyses. (**B**) Effect of IL1A knockdown on cell viability of SiHa. (**C**) IL1A transcript levels of SiHa ctrl-sh-ctrl, SiHa ctrl-sh-p16#1, SiHa ctrl-sh-p16#2, SiHa IL1A-sh-p16#1 and SiHa IL1A-sh-p16#2 were determined by Taqman qRT-PCR analyses. (**D**) IL1A overexpressing affected the cell viability of p16 silencing SiHa.

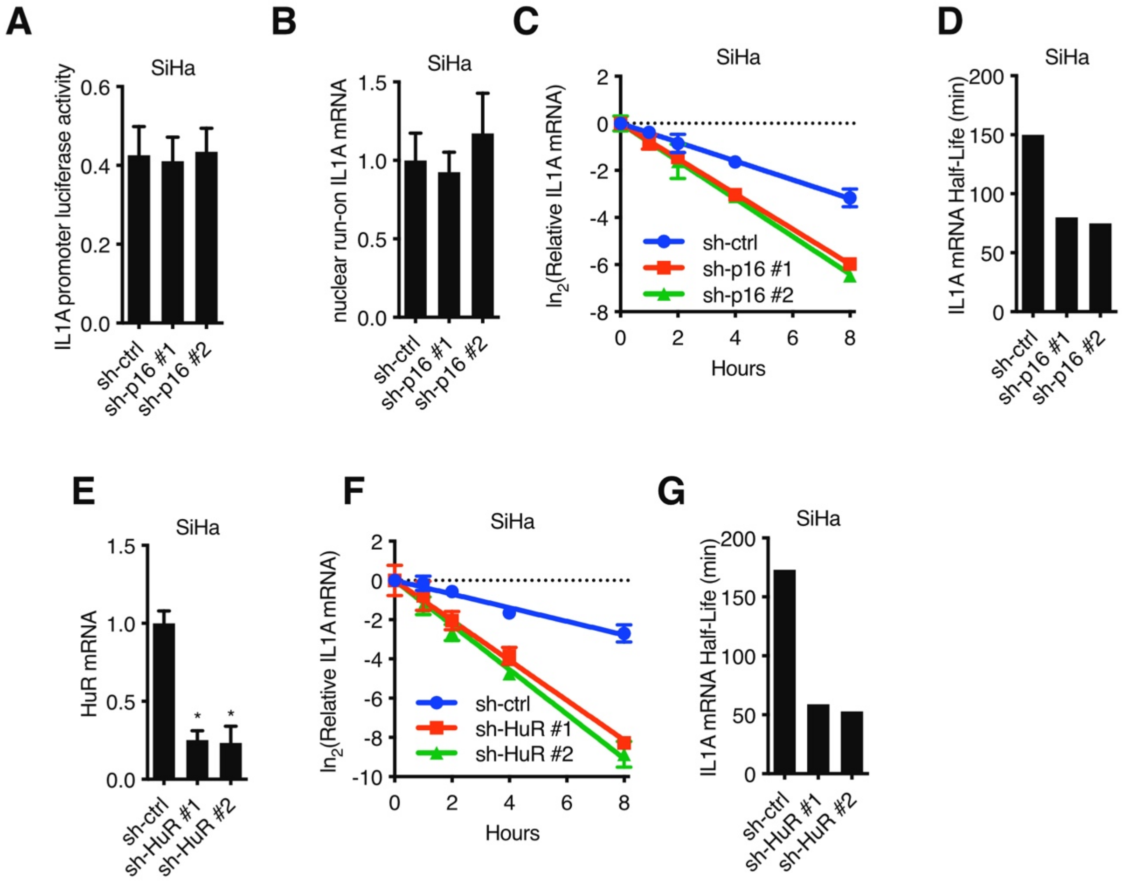

Figure S3. p16-HuR regulated IL1A mRNA stability: (A) The activity of IL1A promoter in SiHa sh-ctrl, SiHa sh-p16#1 and SiHa sh-p16#2 was analyzed by luciferase-based reporter assay. (B) Nuclear run-on assay of SiHa sh-ctrl, SiHa sh-p16#1 and SiHa sh-p16#2. (C) The stability of endogenous IL1A mRNA was influenced by p16 knockdown. (D) The half-life of IL1A mRNA in SiHa sh-ctrl, SiHa sh-p16#1 and SiHa sh-p16#2 was calculated. (E) HuR transcript levels of SiHa sh-ctrl, SiHa sh-HuR#1 and SiHa sh-HuR#2 were determined by SYBR Green qRT-PCR analyses. (F) The stability of endogenous IL1A mRNA was affected by HuR knockdown. (G) The half-life of IL1A mRNA in SiHa sh-ctrl, SiHa sh-HuR#1 and SiHa sh-HuR#2 was calculated.

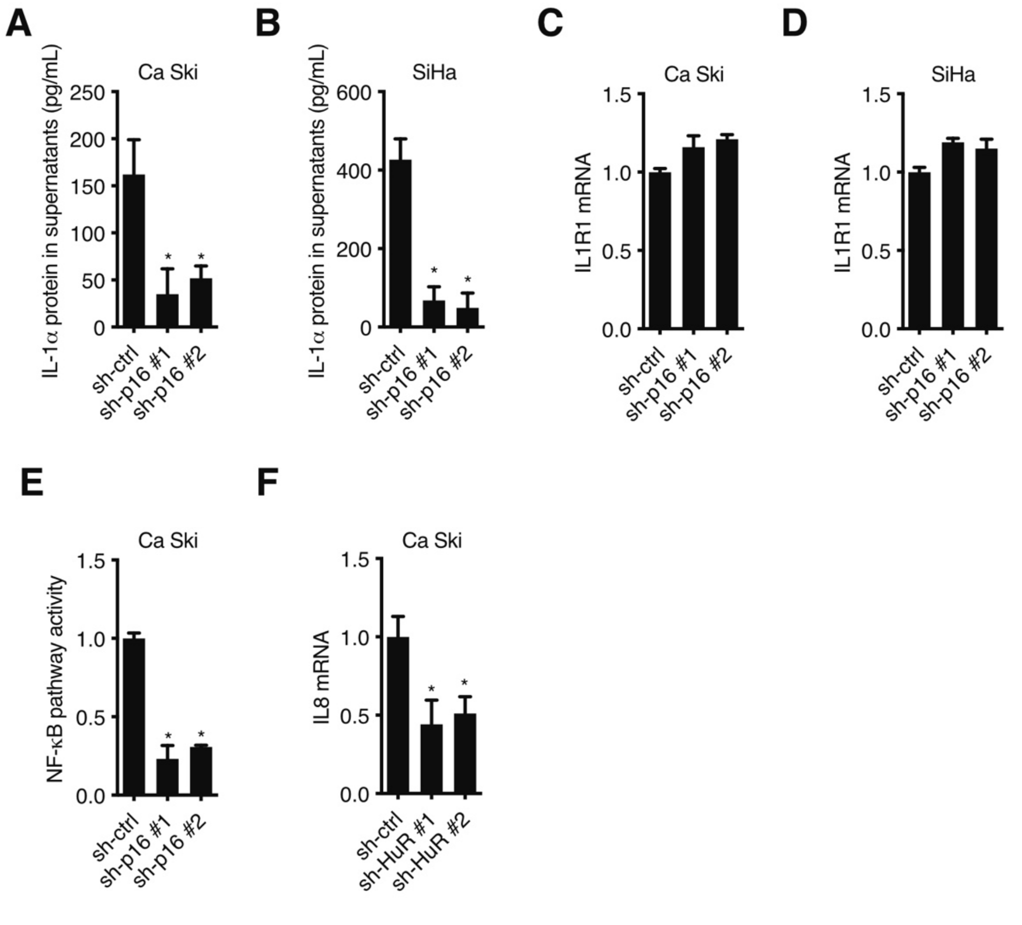

Figure S4. IL1A regulated IL8 through NF-κB pathway: (A) IL-1α protein in supernatants of Ca Ski sh-ctrl, Ca Ski sh-p16#1 and Ca Ski sh-p16#2 were determined by ELISA. (B) IL-1α protein in supernatants of SiHa sh-ctrl, SiHa sh-p16#1 and SiHa sh-p16#2 were determined by ELISA. (C) IL1R1 transcript levels of Ca Ski sh-ctrl, Ca Ski sh-p16#1 and Ca Ski sh-p16#2 were determined by SYBR Green qRT-PCR analyses. (D) IL1R1 transcript levels of SiHa sh-ctrl, SiHa sh-p16#1 and SiHa sh-p16#2 were determined by SYBR Green qRT-PCR analyses. (E) The activity of NF-κB pathway activity in Ca Ski sh-ctrl, Ca Ski sh-p16#1 and Ca Ski sh-p16#2 was analyzed by luciferase-based reporter assay. (F) IL8 transcript levels of Ca Ski sh-ctrl, Ca Ski sh-HuR#1 and Ca Ski sh-HuR#2 were determined by SYBR Green qRT-PCR analyses.

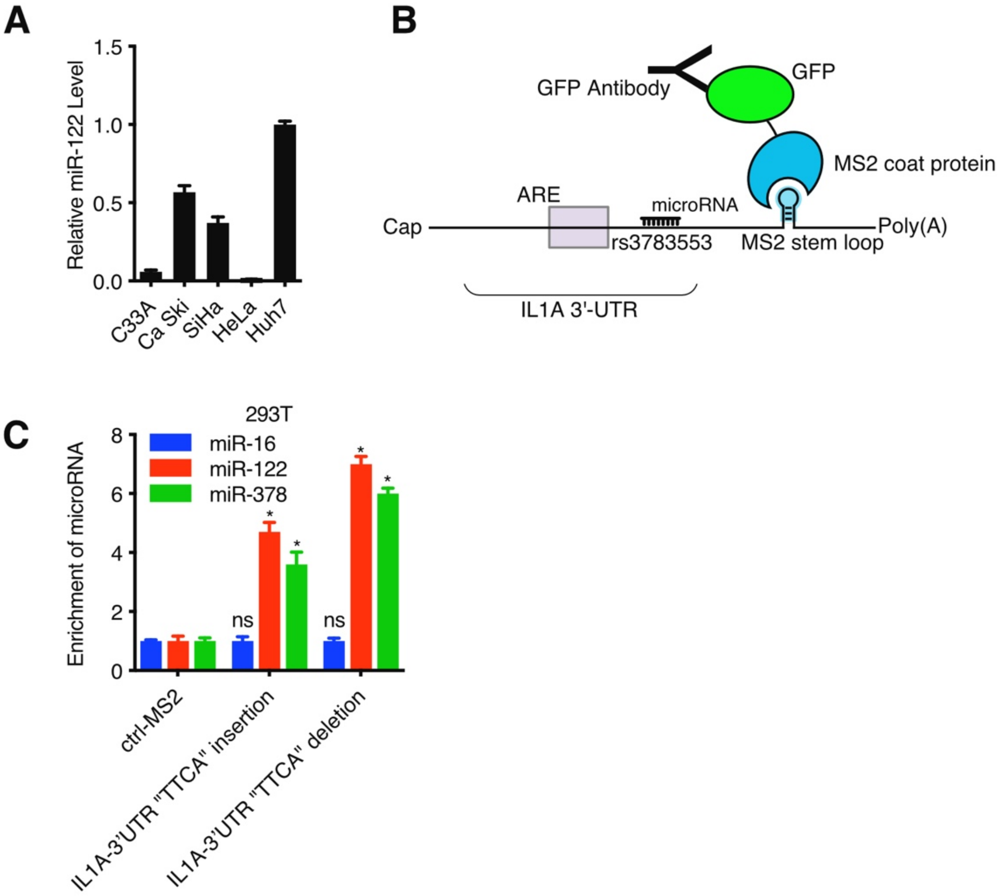

Figure S5. rs3783553 increased IL1A expression level by eliminating miR-122 and miR-378: (A) miR-122 levels of C33A, Ca Ski, SiHa, HeLa, and Huh7 were determined by qRT-PCR analyses. (B) Schematic diagram of MS2 tagging based RNA immunoprecipitation assay. (C) The enrichment of miR-122 and miR-378 after immunoprecipitate were analyzed by qRT-PCR and normalized to U6. miR-16 served as the control. The enrichment of miRNAs was represented as means ± SD (n=3).
